# An abattoir study of the prevalence of foot lesions and claw measurements in water buffalo in Egypt

**DOI:** 10.1186/s12917-024-03877-4

**Published:** 2024-01-20

**Authors:** Shebl E. Salem, Walid Refaai, Mustafa Abd EL Raouf, Mohamed A. Hamed, Shimaa A. Ezzeldein, Eslam F. Eisa, Ayman Mesalam, Thomas W. Maddox, Ahmed Monir

**Affiliations:** 1 Department of Surgery, Anaesthesiology, and Radiology, Faculty of Veterinary Medicine, Zagazig University, Zagazig, 44519 Egypt; 2 Department of Surgery, Anaesthesiology, and Radiology, Faculty of Veterinary Medicine, Aswan University, Aswan, 81528 Egypt; 3 Department of Theriogenology, Faculty of Veterinary Medicine, Zagazig University, Zagazig, 44519 Egypt; 4 Department of Small Animal Clinical Science, Institute of Infection, Veterinary and Ecological Sciences, University of Liverpool, Neston, UK

**Keywords:** Foot Lesions, Claw measurements, Water buffalo, Computed tomography, Ultrasonography, Trimming

## Abstract

**Background:**

Lameness has been associated with compromised animal welfare and reduced productivity in dairy cattle herds worldwide. However, little is known about the prevalence of claw lesions in the dairy buffalo population in Egypt. Furthermore, the optimum measurements for claw trimming in buffalo are unknown. A cross-sectional cadaver study was conducted where 135 pair buffalo hind feet were collected from 4 slaughterhouses and examined for the presence of claw lesions. The proportion and associated 95% confidence interval (CI) of each type of lesion were calculated. A separate set of healthy claws (*n* = 26) underwent ultrasonography (US) and computed tomography (CT). The agreement between US and CT measurements was assessed using Passing-Bablok regression and intraclass correlation coefficient. The CT measurements were used to calculate trimming recommendations.

**Results:**

At least one lesion was identified in 242 claws (89.6%, 95% CI = 85.4–93.0). In healthy claws, poor to moderate agreement was identified between US and CT measurements which could be due a sample size of the study. The average ± standard deviation (SD) minimum recommended external wall length of the lateral and medial claws in heifers was 7.1 ± 0.36 cm and 7.5 ± 0.35 cm, respectively. The average ± SD minimum recommended external wall length in buffaloes over five years of age was 8.2 ± 0.27 cm and 8.4 ± 0.39 cm for the lateral and medial claws, respectively.

**Conclusions:**

The study found a high prevalence of claw lesions in buffalo in Egypt, the clinical significance of which requires further elucidation. Recommended measurements will help guide claw trimming in buffalo to minimise lesions.

**Supplementary Information:**

The online version contains supplementary material available at 10.1186/s12917-024-03877-4.

## Background

Buffalo production is important for food security in Egypt, contributing approximately 28% and 38% of the raw milk and red meat production, respectively [1]. The official buffalo population in Egypt was 1,348,000 heads in 2020 [1]. Historically, buffaloes have been raised under a mixed crop-livestock farming system [2]. However, commercial buffalo farms with semi-intensive and intensive modern production systems have become increasingly available in Egypt in recent years [3, 4]. Most studies on buffaloes in Egypt have focused on endemic infectious diseases, such as foot and mouth disease (FMD) and tropical theileriosis [5–7], or on improving reproductive performance and productivity, such as studying the usefulness of crossbreeding with Italian Mediterranean buffalo [4, 8, 9].

Lameness is associated with reduced welfare and impaired productivity in dairy cattle worldwide [10, 11]. A recent study in Egypt conducted on 55 commercial dairy cattle farms reported an average within-herd lameness prevalence of 43.1% and a 100% herd-level prevalence [12]. Relatively little research has been conducted to investigate the impact of lameness on the buffalo population worldwide. A study by Guccione et al. [13] reported foot lesions in 229 of 1297 (17.7%) multiparous Italian Mediterranean buffaloes subjected to routine trimming on four free-stall dairy buffalo farms in Italy. These lesions were associated with clinical lameness in 206 buffaloes (90.0% of animals with lesions; 15.9% overall). Earlier studies have reported minimal impact of lameness on dairy buffaloes [14, 15]. These studies reported zero prevalence of lameness in three dairy buffalo farms in Italy and attributed this reduced prevalence to a lack of genetic predisposition to lameness and the lower feeding regimen in buffalo compared with cattle. In Egypt, no previous study has documented foot lesions in buffaloes or investigated the prevalence of lameness in this population of animals. FMD, a leading infectious cause of foot lesions in cattle and buffalo, is endemic in Egypt, with multiple outbreaks reported annually [16]. The perceived low impact of lameness in dairy buffaloes and the presence of other endemic, economically important diseases to which research resources have been allocated could be the reason why lameness research in buffaloes has not drawn much attention.

Routine claw trimming has been established as a component of a lameness prevention plan for dairy cattle herds [17–20], with 82.4% of dairy farmers practicing routine trimming in the UK [21]. Functional claw trimming reduces the risk of lameness development by improving foot balance between the lateral and medial claws, increasing the contact area with the ground [22], resulting in improved grip [23], and reducing external weight from the typical sole ulcer site at the axial sole [17, 24]. It also contributes to the early detection and treatment of subclinical claw lesions before they develop into clinical lameness [19]. A lack of routine trimming has been associated with claw horn disruption lesions, such as white line fissures and sole ulceration [25].

Because of the high propensity for over-trimming, proper claw trimming is equally as important as preventive trimming itself [25]. Trimming to a minimum sole thickness of 5 mm at the tip of the 3rd phalanx has been standardized in dairy cattle to prevent compression damage to the corium, particularly at the level of the flexor tubercle, and to prevent the development of a thin sole [25–27]. Furthermore, trimming the claws to an average length of 7.2 – 9 cm has been recommended to achieve a 5 mm minimal sole thickness in dairy cattle [25, 27, 28]. Over-trimming can result in a thin sole with subsequent compression of the subsolar soft tissue and the development of claw lesions such as toe ulcers [29]. These claw measurement recommendations are lacking for buffaloes. Therefore, the objectives of the current study were to 1) document foot lesions in buffalo hind feet collected from local abattoirs, 2) use data obtained from computed tomography (CT) examination to standardize claw-trimming measurements in Egyptian buffalo, 3) investigate the utility of ultrasonography as an objective method to measure the sole and solar soft tissue thickness at the ground surface of the hind claws, and 4) investigate the agreement between measurements taken by CT and ultrasonography examination.

## Results

During the study period (November –December 2019), we examined 270 hind feet of 135 buffaloes. They were recruited from four abattoirs located in three Egyptian governorates (Cairo, Al Sharqiya, and Dakahlia). The feet were examined during 8 visits to these abattoirs. The buffaloes examined included 62 males (45.9%; 95% confidence interval [CI] = 37.3 – 54.7) and 73 females (54.1%; 95% CI = 45.3 – 62.7). The age distribution of examined animals was unknown, however, fattened male buffaloes are generally slaughtered at 2–3 years of age and by law, female buffaloes are not slaughtered before 5 years of age. At least one lesion was identified in 242 feet (89.6%, 95% CI = 85.4 – 93.0). The maximum number of lesions identified in a single foot was 11, which were identified in four feet (1.5%, 95% CI = 0.41 – 3.8). Table 1 presents the lesions identified, their prevalence, and associated 95% CIs.

**Table 1:** Lesions identified, their prevalence and associated 95% confidence intervals in 270 hind buffalo feet examined in 4 local abattoirs in Egypt

Lesion	Affected Claw	Number of feet affected	Proportion	95% CI of percentage	
				Lower	Upper
Asymmetrical claw	-	2	0.74	0.09	2.7
Concave dorsal wall	-	2	0.74	0.09	2.7
Corkscrew claws	-	7	2.6	1.1	5.3
Scissors claws	-	65	24.1	19.1	29.6
Digital dermatitis	-	0	0	0	0
Interdigital dermatitis	-	84	31.11	25.6	37.0
Interdigital hyperplasia	-	3	1.11	0.23	3.21
Interdigital phlegmon	-	0	0	0	0
Swelling of the coronet	-	0	0	0	0
Heel horn erosion	-	112	41.5	35.5	47.6
Axial fissure	LC & MC	0	0	0	0
Horizontal fissure	LC	1	0.37	0.01	2.1
	MC	3	1.1	0.23	3.2
Vertical fissure	LC & MC	0	0	0	0
Diffuse sole Haemorrhage	LC	14	5.19	2.86	8.55
	MC	10	3.7	1.79	6.71
Circumscribed (Localized) sole Haemorrhage	LC	7	2.59	1.05	5.27
	MC	6	2.22	0.82	4.77
Sole ulcer	LC	1	0.37	0.01	2.05
	MC	0	0	0	0
Toe ulcer	LC & MC	0	0	0	0
Heel ulcer	LC & MC	0	0	0	0
Toe necrosis	LC & MC	0	0	0	0
Thin sole	LC	6	2.2	0.82	4.8
	MC	4	1.5	0.41	3.8
White line fissure	LC	21	7.8	4.9	11.6
	MC	6	2.22	0.82	4.8
White line abscess	LC & MC	0	0	0	0
Double sole	LC	53	19.6	15.1	24.9
	MC	44	16.3	12.1	21.3

*LC* lateral claw, *MC* medial claw

**Fig. 1:**
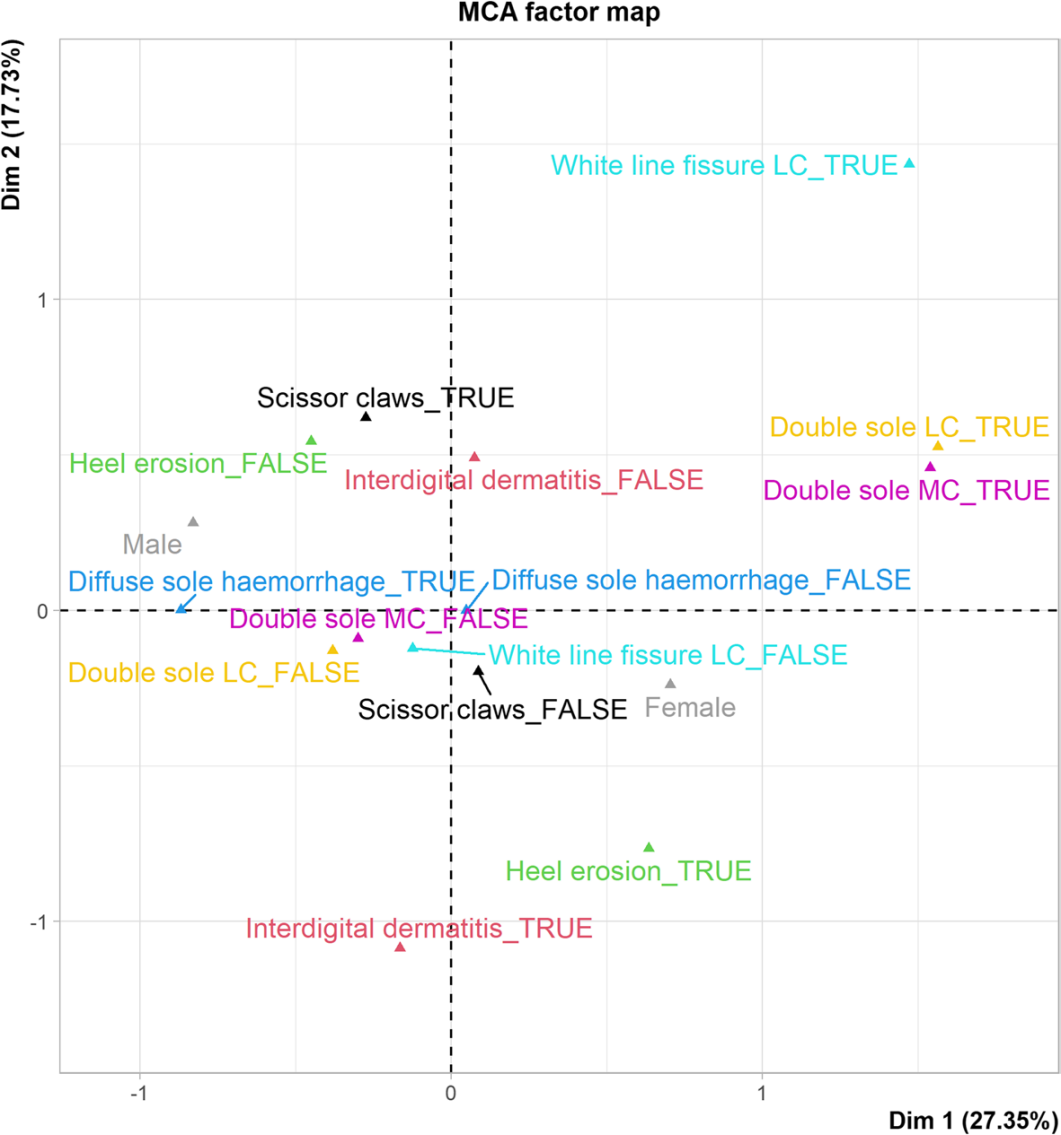
Multiple correspondence analysis of the relationship between sex of the buffalo (a proxy for age) and claw lesions identified at ≥ 5% prevalence. The first two dimensions explained 45.1% of variation in the data. There is correlation between double sole, heel erosion, white line fissure, interdigital dermatitis, and female buffaloes and between scissor claws and sole haemorrhage and male buffaloes

The association between lesions identified at ≥ 5% prevalence (scissor claws, interdigital dermatitis, heel horn erosion, diffuse sole haemorrhage, white line fissure, double sole) and sex of the animals (male or female) were explored using multiple correspondence analysis, and the results are presented in Fig. 1. The first two dimensions explained 45.1% of the total variation in the data. There was a strong association between the presence of heel horn erosion, double sole, white line fissure, and female buffaloes. In contrast, male buffaloes were strongly correlated with scissor claws and diffuse sole haemorrhage.

**Fig. 2:**
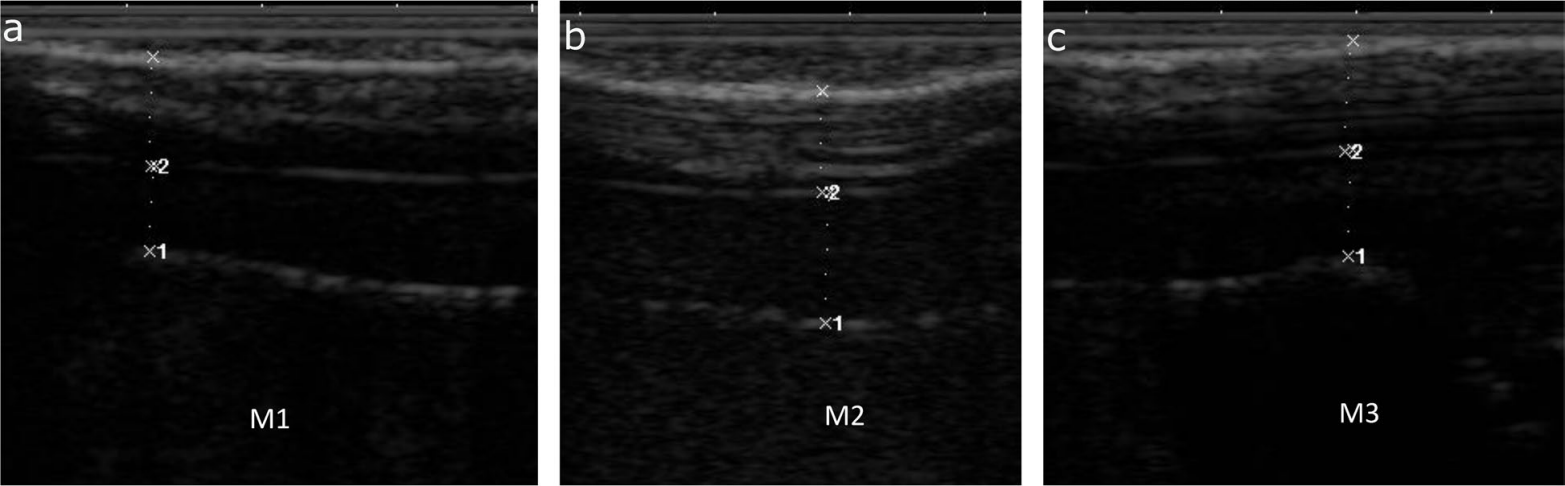
Ultrasonography image obtained from the ground surface of the claw showing locations of measurements taken for the corium and sole thickness. The 3 hyper-reflective lines from the top down are the sole ground surface, sole-corium junction, and the ventral surface of the 3rd phalanx. Location M1 is at the most apical margin of the 3rd phalanx, M2 is at the deepest concavity of the 3rd phalanx and M3 is at the region of the flexor tubercle

**Fig. 3:**
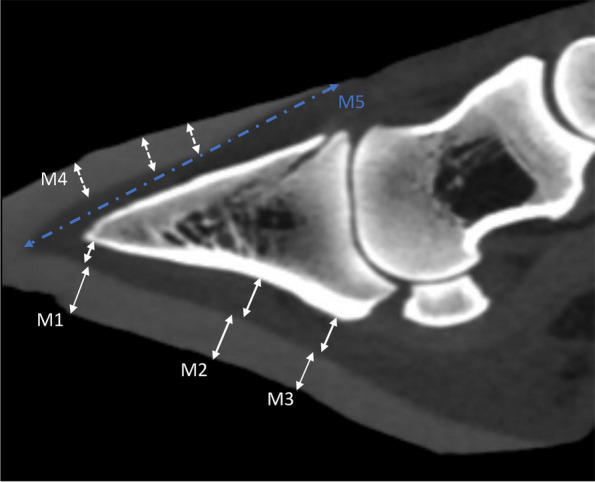
Approximately mid-sagittal computed tomography image of a boffola hind claw. The figure shows the locations of measurements taken. M1 is at the most apical margin of 3rd phalanx, M2 is at the deepest concavity of the 3rd phalanx, M3 is at the region of the flexor tubercle, M4 is the dorsal wall thickness measured at 3 different locations along the dorsal surface of the 3rd phalanx, M5 is the internal wall length measured from the most proximal border of the wall to the tip of the wall corium

Healthy claws from buffalo heifers (*n* = 14) and buffaloes > 5 years of age (*n* = 12) underwent US and CT examinations. Summary statistics of claw measurements obtained and the results of the Shapiro–Wilk test of normal distribution are presented in Table 2. Measurements obtained using ultrasonography (M1, M2, M3) and CT (M1, M2, M3, M4, M5, claw angle) are presented for the two study groups and for the medial and lateral claws separately Figs. 2 and 3. Some of the results obtained did not follow a normal distribution (*P* < 0.05). The mean ± standard deviation (SD) of dorsal wall thickness in the old age group was 8.1 ± 0.72 and 8.6 ± 1.1 mm for the lateral and medial claws, respectively. In heifers, the mean dorsal wall thickness was 6.6 ± 0.34 and 6.9 ± 0.38 for the lateral and medial claws, respectively. The mean internal wall length (measure M5) was 7.4 ± 0.23 cm and 7.5 ± 0.34 cm for the lateral and medial claws, respectively in the old age group and 6.6 ± 0.34 and 6.9 ± 0.38 for the lateral and medial claws, respectively in heifers.

**Table 2:** Summary statistics and results of the Shapiro–Wilk test for normality for measurements obtained from ultrasonography and computed tomography examination of 26 buffalo hind feet

Variable		Mean	SD	Median	1st quartile	3rd quartile	Range	Shapiro–Wilk test *p* value
**Result of USG examination (buffalo heifers)**								
Corium M1 (mm)	LC	4.5	1.3	4.4	4.0	5.2	2.0 – 6.8	0.1
	MC	4.0	0.98	4.1	3.4	4.5	1.7 – 6.4	0.2
Sole M1 (mm)	LC	4.5	0.72	4.5	4.1	5.0	2.9 – 5.9	0.8
	MC	4.9	1.3	5.0	4.2	5.5	2.7 – 9.3	0.002
Corium M2 (mm)	LC	7.2	1.9	7.6	5.9	8.4	3.6 – 10.6	0.1
	MC	7.1	2.1	7.0	5.5	8.5	3.5 – 10.5	0.04
Sole M2 (mm)	LC	4.8	1.5	4.7	4.1	5.1	2.5 – 9.4	< 0.001
	MC	4.5	1.3	4.3	3.8	4.9	2.3 – 8.8	< 0.001
Corium M3 (mm)	LC	6.4	1.05	6.3	5.7	7.1	4.0 – 8.3	0.6
	MC	6.3	1.1	6.3	5.4	7.3	4.0 – 8.4	0.2
Sole M3 (mm)	LC	5.4	1.6	5.5	4.6	6.2	2.8 – 9.3	0.2
	MC	5.0	1.4	4.6	4.0	5.8	2.6 – 7.9	0.03
**Result of USG examination (old buffalo group)**								
Corium M1 (mm)	LC	4.8	1.2	4.3	3.7	5.7	3.1 – 7.0	0.1
	MC	4.8	1.2	4.6	3.8	5.7	3.0 – 6.7	0.2
Sole M1 (mm)	LC	6.0	1.4	6.0	5.0	6.7	4.1 – 8.7	0.3
	MC	7.5	1.7	7.4	5.9	8.7	5.3 – 11.1	0.06
Corium M2 (mm)	LC	7.8	2.1	8.4	6.0	8.8	4.3 – 11.2	0.2
	MC	8.4	1.4	8.6	7.3	9.6	5.9 – 10.5	0.2
Sole M2	LC	6.9	1.6	7.6	5.4	8.3	4.5 – 8.8	0.002
	MC	7.1	1.7	7.5	5.4	7.8	4.6 – 10.2	0.07
Corium M3	LC	7.0	1.5	6.5	6.0	8.3	4.2 – 9.4	0.2
	MC	6.9	1.4	6.9	5.5	8.2	5.2 – 9.1	0.03
Sole M3	LC	7.0	1.8	7.4	5.4	8.1	3.6 – 10.2	0.2
	MC	6.5	2.1	5.9	5.1	7.8	3.5 – 10.3	0.2
**Results of CT examination (buffalo heifers)**								
Corium M1 (mm)	LC	3.5	0.67	3.4	3.0	3.7	2.5 – 5.5	< 0.001
	MC	3.1	0.78	3.0	2.7	3.6	1.5 – 4.8	0.5
Sole M1 (mm)	LC	6.8	2.0	6.2	5.1	7.8	4.4 – 11.2	< .001
	MC	7.8	2.0	8.3	6.0	9.1	4.4 – 11.3	0.1
Corium M2 (mm)	LC	7.5	1.2	7.8	7.1	8.3	4.8 – 10.0	0.002
	MC	6.4	1.1	6.5	6.0	7.1	4.1 – 8.5	0.1
Sole M2 (mm)	LC	5.9	2.7	4.6	3.9	7.2	3.1 – 11.9	< 0.001
	MC	6.9	2.8	6.8	4.6	10.1	2.3 – 12.3	0.03
Corium M3 (mm)	LC	6.8	0.86	6.8	6.3	7.4	5.0 – 8.4	0.7
	MC	6.3	1.0	6.3	5.6	6.7	4.3 – 8.9	0.2
Sole M3 (mm)	LC	6.1	2.0	5.9	4.2	7.0	3.0 – 10.6	0.01
	MC	7.1	2.5	7.4	5.3	9.2	2.3 – 11.1	0.2
Dorsal wall M4 (mm)	LC	5.4	0.69	5.5	4.8	5.8	3.9 – 6.7	0.6
	MC	5.9	0.82	5.8	5.4	6.3	4.7 – 8.5	0.001
Internal wall length M5 (cm)	LC	6.6	0.34	6.7	6.3	6.8	6.1 – 7.1	0.3
	MC	6.9	0.38	7.0	6.8	7.2	6.3 – 7.6	0.9
Angle	LC	48.1	1.1	48.0	47.5	48.5	46.5 – 50.0	0.5
	MC	48.8	2.9	49.0	48.0	50.0	43.0 – 54.8	0.8
“a” segment (mm)	LC	4.9	0.70	4.8	4.37	5.46	3.3 – 6.1	0.3
	MC	5.2	0.76	5.1	4.7	5.6	3.9 – 7.3	0.2
“c” segment (mm)	LC	6.7	0.12	6.7	6.7	6.7	6.5 – 6.9	0.01
	MC	6.7	0.29	6.6	6.5	6.7	6.1 – 7.3	0.01
**Results of CT examination (old buffalo group)**								
Corium M1 (mm)	LC	3.8	0.74	3.8	3.1	4.5	2.7 – 5.0	0.03
	MC	3.3	0.87	3.2	2.9	3.5	2.2 – 5.5	0.01
Sole M1 (mm)	LC	10.3	2.5	10.0	9.5	10.6	6.1 – 15.4	0.01
	MC	10.6	3.2	10.3	9.3	11.7	5.3 – 16.9	0.1
Corium M2 (mm)	LC	8.6	1.4	8.7	8.3	9.3	4.7 – 10.5	< 0.001
	MC	7.7	1.1	7.5	7.1	8.4	5.7 – 10.1	0.2
Sole M2 (mm)	LC	10.6	2.7	10.0	8.8	11.7	6.2 – 16.6	0.03
	MC	10.6	3.1	10.7	9.1	11.5	5.2 – 17.4	0.02
Corium M3 (mm)	LC	8.0	1.1	8.3	7.4	8.9	5.8 – 9.9	0.3
	MC	6.8	0.91	6.9	6.1	7.5	5.2 – 8.3	0.5
Sole M3 (mm)	LC	9.5	2.1	9.0	8.4	10.6	6.3 – 13.8	0.03
	MC	8.3	2.4	7.8	6.4	10.5	5.2 – 12.0	0.03
Dorsal wall M4 (mm)	LC	8.1	0.72	7.9	7.5	8.6	7.0 – 9.6	0.2
	MC	8.6	1.1	8.5	7.9	9.5	6.8 – 11.2	0.6
Internal wall length M5 (cm)	LC	7.4	0.23	7.5	7.2	7.6	7.2 – 7.8	0.4
	MC	7.5	0.34	7.6	7.4	7.7	6.7 – 7.9	0.1
Angle	LC	46.6	3.9	45.5	43.8	48.6	42.2 – 55.0	0.2
	MC	45.3	4.3	45.0	44.2	46.7	38.0 – 54.0	0.6
“a” segment (mm)	LC	7.7	1.1	7.6	7.3	8.4	5.4 – 10.2	0.5
	MC	8.6	0.76	8.7	7.7	9.2	6.1 – 11.6	0.9
“c” segment (mm)	LC	6.9	0.40	7.0	6.6	7.3	6.1 – 7.4	0.1
	MC	7.1	0.50	7.1	6.9	7.2	6.2 – 8.1	0.01

**Fig. 4:**
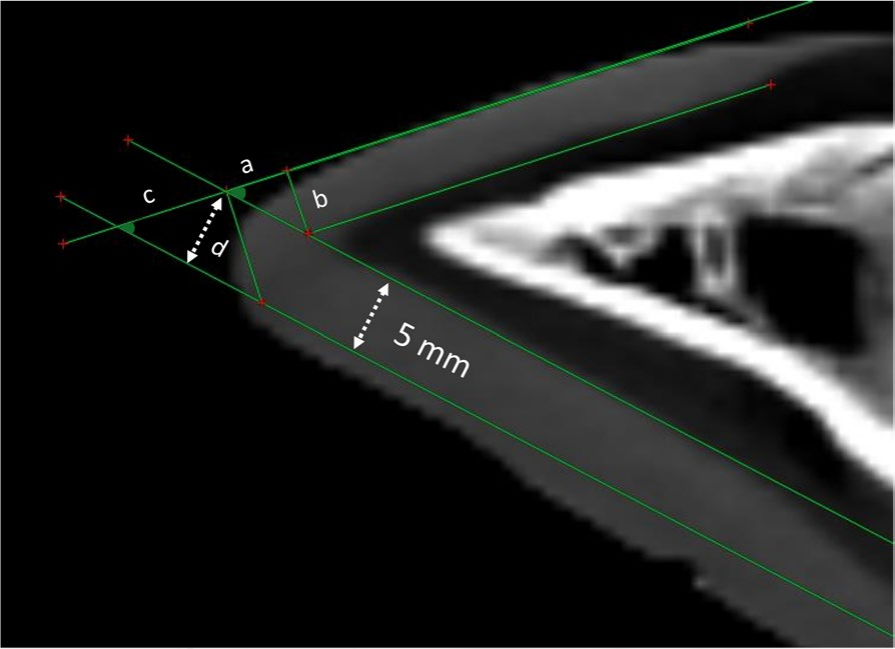
Calculation of the recommended minimal external wall length. Segment “a” is added to the internal wall length if the claw is trimmed to a step and segment “a + c” is added if the claw is trimmed to a point. Segment “a” is calculated as a = b / (tangent of claw angle) where “b” is the dorsal wall sickness. Segment “c” is calculated as c = d / (sine claw angle) where “d” is the minimum recommended sole thickness of 5 mm at the tip of the 3rd phalanx

*M1* measurements obtained at the apex of the 3rd phalanx, *M2* measurements obtained at the most concave portion of the 3rd phalanx, *M3* measurements obtained at the level of the flexor tubercle, *M4* is the dorsal wall thickness, *M5* is the internal wall length from the proximal border of the wall to the tip of the wall corium, *claw angle* is the angle made by two straight lines one parallel to the dorsum of the 3rd phalanx and the other to the ground border of the sole. “a” segment is added to the internal wall length if the claws are trimmed to a step. “a” and “c” segments are added to the internal wall length if the claws are trimmed to a point (see Fig. 4).  *LC* lateral claw, *MC* medial claw

The internal wall length measured in the CT studies was adjusted to calculate the minimal external wall length if the claws were trimmed to a point or a step. This considered the dorsal wall thickness, claw angle, and minimum sole thickness of 5 mm at the tip of 3rd phalanx (Fig. 4). These calculations indicated that the average ± SD minimum external wall length of the lateral and medial claws in the heifers should be 7.1 ± 0.36 cm and 7.5 ± 0.35 cm, respectively if the claws are trimmed to a step. These measurements increased to a mean ± SD of 7.7 ± 0.37 cm and 8.2 ± 0.34 cm for the lateral and medial claws, respectively, if the claws are trimmed to a point in the same age group. In the old age group, the average minimum recommended external wall length was 8.2 ± 0.27 cm and 8.4 ± 0.39 cm for the lateral and medial claws, respectively if they are trimmed to a step and 8.9 ± 0.30 cm and 9.1 ± 0.43 cm for the lateral and medial claws, respectively if they are trimmed to a point. The results of the calculations are listed in Table 3.

**Table 3:** Summary statistics and results of Shapiro–Wilk test of normal distribution of calculated recommended minimal dorsal wall length during trimming in buffalo heifers and buffaloes >5 years of age  if the claws are trimmed to step or to a point

**Variable**		**Mean**	**SD**	**Median**	**1st quartile**	**3rd quartile**	**Range**	**Shapiro–Wilk test *p* value**
**Buffalo heifers**								
LC	Trimming to a step	7.1	0.36	7.2	6.7	7.4	6.5 – 7.7	0.004
	Trimming to a point	7.7	0.37	7.9	7.4	8.1	7.2 – 8.4	0.003
MC	Trimming to a step	7.5	0.35	7.5	7.3	7.8	6.8 – 8.1	0.2
	Trimming to a point	8.2	0.34	8.1	8.0	8.4	7.5 – 8.8	0.3
**Buffaloes >5 years of age**								
LC	Trimming to a step	8.2	0.27	8.2	8.0	8.4	7.9 – 8.6	0.005
	Trimming to a point	8.9	0.30	8.9	8.6	9.2	8.5 – 9.3	0.001
MC	Trimming to a step	8.4	0.39	8.5	8.2	8.6	8.2 – 8.8	0.001
	Trimming to a point	9.1	0.43	9.1	8.9	9.4	7.9 – 9.6	0.001

*LC* lateral claw, *MC* medial claw

**Fig. 5:**
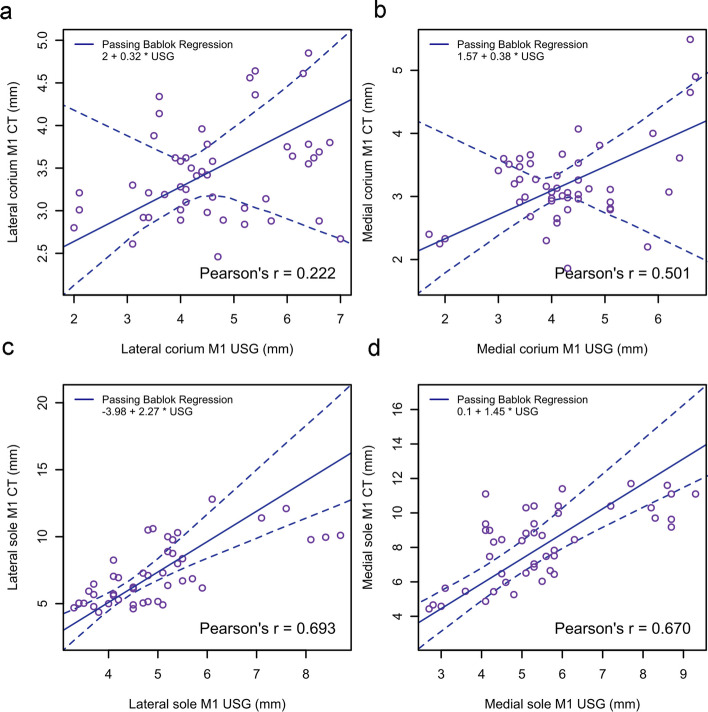
Passing–Bablok regression plot for corium (**a**, **b**) and sole (**c**, **d**) thicknesses obtained from ultrasonography and computed tomography at the most apical margin of the 3rd phalanx (location M1). The thick blue line is the line of best fit and the dashed lines are 95% confidence interval. The intercept, slope and Pearson’s correlation coefficient are demonstrated on the plot

**Fig. 6:**
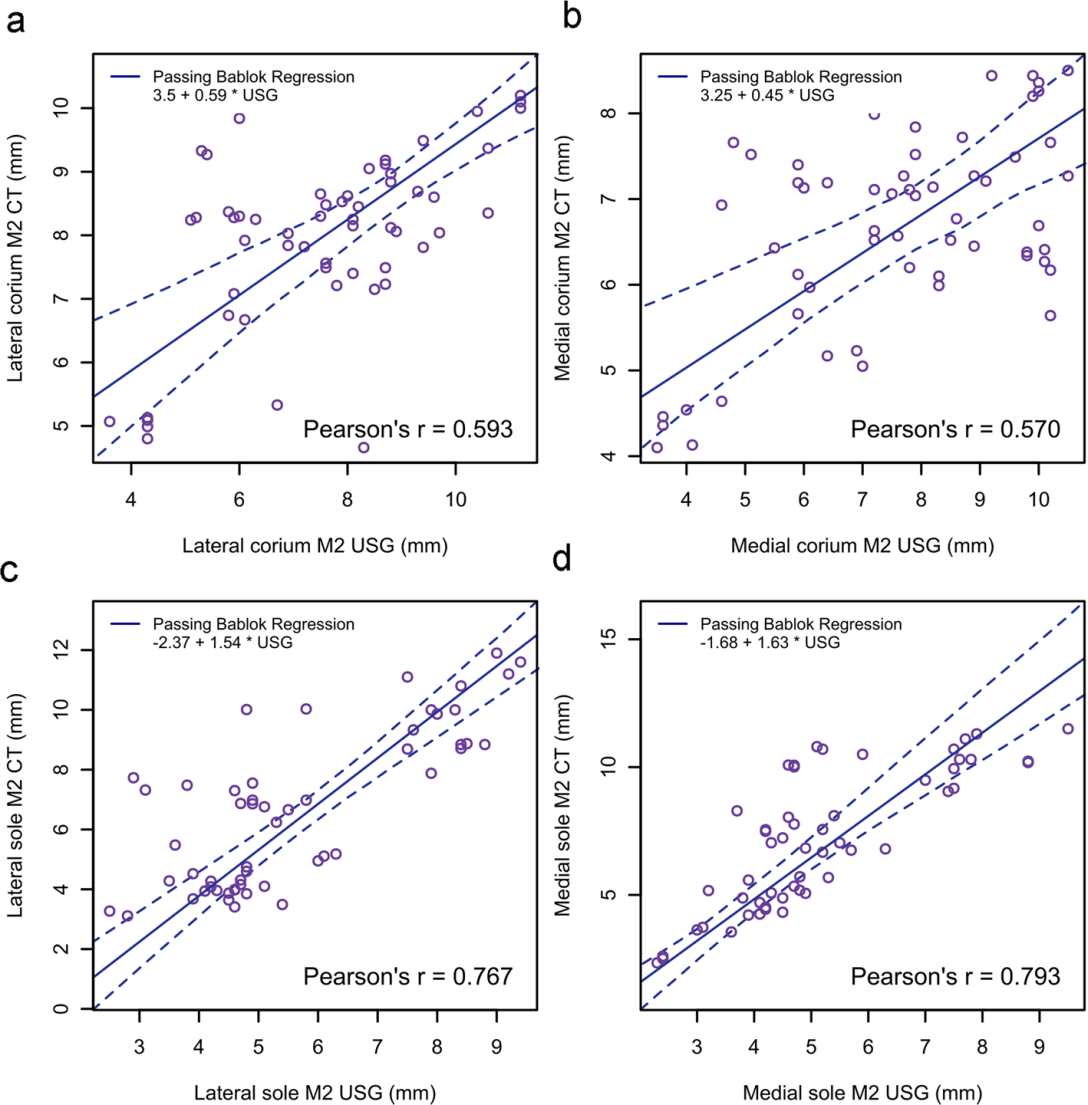
Passing–Bablok regression plot for corium (**a**, **b**) and sole (**c**, **d**) thicknesses obtained from ultrasonography and computed tomography at the deepest concavity of the 3rd phalanx (location M2). The thick blue line is the line of best fit and the dashed lines are 95% confidence interval. The intercept, slope and Pearson’s correlation coefficient are demonstrated on the plot

**Fig.7:**
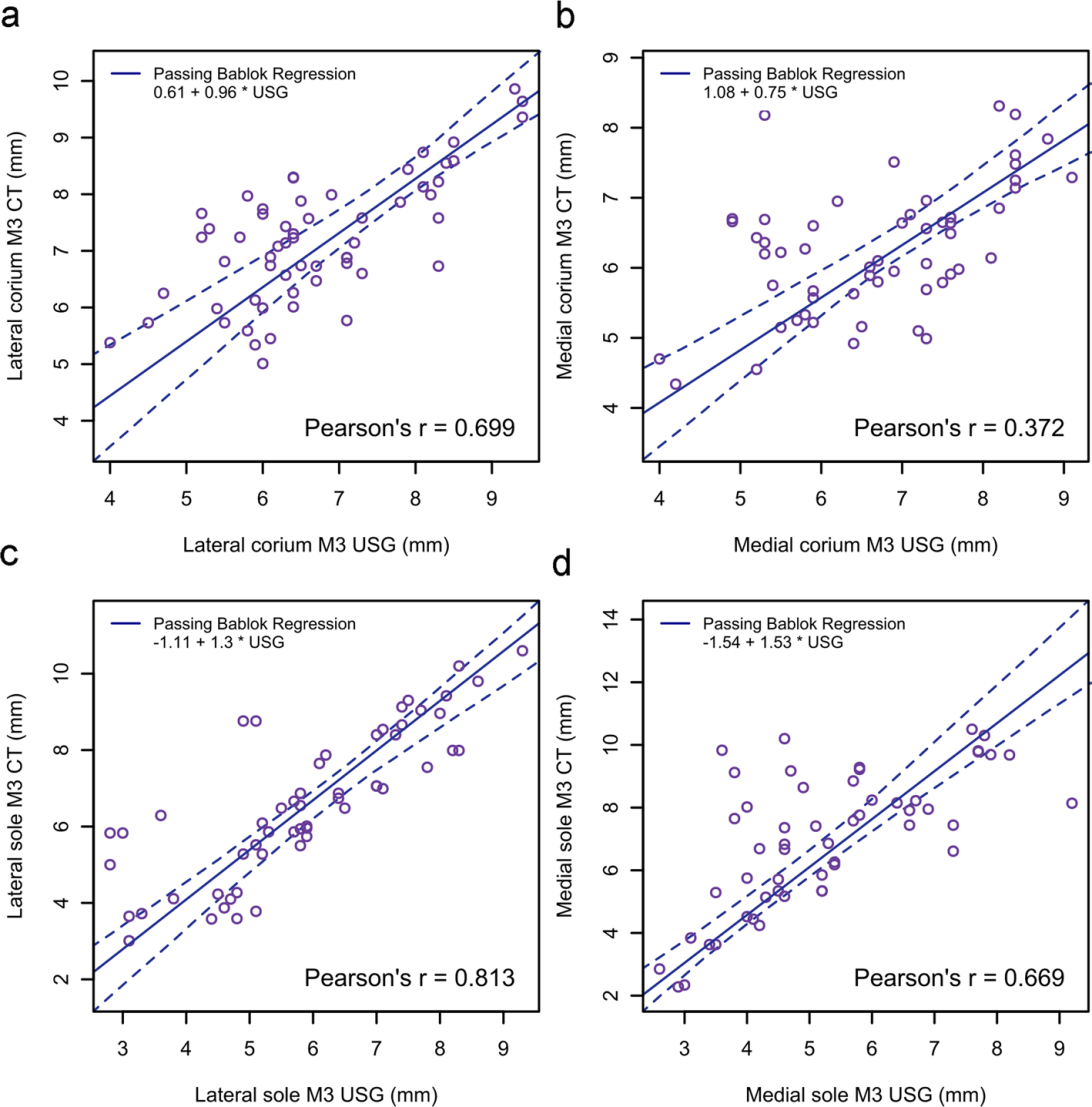
Passing–Bablok regression plot for corium (**a**, **b**) and sole (**c**, **d**) thickness obtained from ultrasonography and computed tomography at the flexor tubercle of the 3rd phalanx (location M3). The thick blue line is the line of best fit and the dashed lines are 95% confidence interval. The intercept, slope and Pearson’s correlation coefficient are demonstrated on the plot

The results of the Passing–Bablok regression and ICC for the agreement between measurements taken using ultrasonography and CT are presented in Table 4 and Figs. 5, 6 and 7. There was poor to moderate agreement between measurements.

**Table 4:** Results of the Passing–Bablok regression of the agreement between measurements taken by USG examinations and CT studies

Measures		Intercept	CI of the intercept^a^	Slope	CI of the slope^a^	ICC	ICC CI^b^
LC M1	Corium	2	1.1, 4.7	0.32	-0.30, 0.55	0.11	-0.08, 0.32
	Sole horn	-4	-8.6, -0.50	2.3	1.5, 3.4	0.34	-0.10, 0.66
MC M1	Corium	1.6	0.53, 4.8	0.40	-0,41, 0.62	0.27	-0.07, 0.55
	Sole horn	0.1	-2.8, 1.7	1.5	1.1, 2.1	0.33	-0.10, 0.67
LC M2	Corium	3.5	2.1, 5.3	0.60	0.40, 0.80	0.55	0.34, 0.71
	Sole horn	-2.4	-3.90, -0.65	1.54	1.30, 1.80	0.67	0.38, 0.82
MC M2	Corium	3.3	2.2, 4.9	0.45	0.27, 0.60	0.42	0.14, 0.63
	Sole horn	-1.7	-3.2, -0.60	1.6	1.4, 1.9	0.52	-.05, 0.79
LC M3	Corium	0.61	-1.3, 2.3	0.96	0.75, 1.2	0.64	0.38, 0.80
	Sole horn	-1.1	-2.6, 0.15	1.3	1.1, 1.5	0.74	0.45, 0.87
MC M3	Corium	1.1	-0.26, 2.2	0.75	0.60, 0.93	0.34	0.12, 0.56
	Sole horn	-1.5	-2.7, -0.3	1.5	1.3, 1.8	0.45	-0.04, 0.73

M1, M2 and M3 are measures taken at the distal tip of the 3rd phalanx, the most concave portion, and the flexor tubercle of the 3rd phalanx, respectively. CI = 95% confidence interval, *ICC* intraclass correlation coefficient

^a^Presence of zero in the CI of intercept, and one in the CI of slope indicate the two methods are comparable

^b^Absence of zero in the CI of ICC indicate rejection of null hypothesis of no agreement

## Discussion

To our knowledge, this is the first study to provide information on claw lesions in the buffalo population in Egypt and to set recommendations about the optimum dorsal wall length of claws during trimming in two different age groups of buffalo. Furthermore, this study evaluated the use of ultrasonography as a cost-effective, non-invasive, and objective technique to guide claw trimming in buffaloes.

The current study reported a very high prevalence of foot lesions (89.6%) in buffalo feet. A study that evaluated post-mortem assessment of claws as a welfare indicator of feedlot cattle at slaughter reported a high prevalence of claw disorders, such as abnormally shaped claws (61%) and claw wall fissures (26.7%) [30], which is consistent with the current study. Hind foot lesions have also been reported to occur at a prevalence of 32% postmortem in another abattoir-based study of feedlot cattle [31]. Although these lesions are typically associated with compromised animal welfare, their clinical significance cannot be assessed using cadaver study designs. Lesions identified during routine trimming of Italian Mediterranean buffaloes (animal-level prevalence of 17.7%) were associated with clinical signs of lameness in 15.9% of the trimmed buffaloes. Further studies are required to assess the clinical significance of foot lesions in Egyptian buffaloes.

In the current study, foot lesions were examined in slaughtered buffalo which included females over 5 years of age and fattening males between 2–3 years of age. We found that sole haemorrhage was associated with feedlot/male buffalo. A study by Magrin et al. [32] reported a sole haemorrhage prevalence of 65% in the claws of veal calves fed high-carbohydrate diets. Another abattoir-based study of feedlot cattle reported that concrete slatted floors were significantly associated with increased risk of sole haemorrhage [33]. We did not collect information on the management practices of buffaloes in the current study; however, the high prevalence of sole haemorrhage could also be due to feeding a high-carbohydrate diet or managing these animals on solid concrete floors, which has been reported as a common management practice in Egypt [3]. Scissor claws were also more prevalent in feedlot/male buffaloes in the current study, which is consistent with previous studies on feedlot cattle [30, 31].

Double sole, heel horn erosion, and white line fissures were more prevalent in female buffaloes over 5 years age. Possible reasons for this relationship include a lack of routine trimming, poor farm hygiene, or previous infections with FMD. Lack of routine trimming has previously been associated with claw horn disruption lesions in cattle [25]. Poor farm hygiene is prevalent in Egypt and could be one possible reason for the increased prevalence of heel erosion observed in the current study [12, 34]. FMD is endemic in Egypt [5] and is frequently associated with the development of ulcerative lesions in the coronary band [35], with subsequent growth of abnormally weak horn, which may result in the development of a double sole [36].

In the current study, we used ultrasonography to determine the sole and solar soft tissue thickness (combined digital cushion and corium thickness) of the hind claws of female buffaloes from two age groups. Images were successfully obtained from all animals using a linear rectal ultrasound probe, consistent with previous studies on cattle [37, 38]. The probe frequency had to be decreased to 4 MHz in claws with greater sole thickness in older buffaloes, which is consistent with the results of Tsuka et al. [39], who reported that better images were obtained at lower ultrasound probe frequencies. Ultrasonography has been validated to provide objective measurements of the claws in cattle, and has been used to study, for example, the effect of changes in sole soft-tissue thickness and/or echogenicity on the development of claw horn disruption lesions [37, 40, 41], to objectively guide claw trimming [39, 42], and to diagnose pedal bone fractures [43]. Here, we confirmed the utility of ultrasonography for examining the claws of buffaloes. Further research utilizing this diagnostic technique in buffaloes is warranted. Solar soft tissue measurements obtained at M2 were consistently greater than those obtained at other locations in both age groups, which agrees with previous cattle studies [39].

The current study reported a poor-to-moderate agreement between measurements obtained by ultrasonography and CT. Kofler et al. [44] reported greater correlation between ultrasound- and CT-measured sole horn thickness (*r* = 0.83 – 0.89) than between ultrasound- and CT-measured solar soft tissue thickness (*r* = 0.51 – 0.64) which was not observed in the present study. A recent study reported strong correlation (*r* = 0.91 – 0.92) between CT- and ultrasound-measured sole horn thickness in cattle [39]. The reduced agreement between measurements obtained by ultrasonography and CT reported here compared to the later study could be due to a small sample size of the present study, in which we examined only 26 hind buffalo feet [45].

In the current study, we used trigonometry to calculate the minimum recommended dorsal wall length, considering the dorsal wall thickness, claw angle, and internal wall length measured using CT and assuming a minimal sole thickness at the apex of 3rd phalanx of 5 mm [27]. In heifers, an average minimum dorsal wall length of 7.5 and 8.2 cm if the claws were trimmed to a step or to a point, respectively was calculated. A study that investigated current practices of preventative claw trimming in UK dairy herds reported that only 5.9% of respondents practised preventative claw trimming in pre-calving heifers [21]. A randomized controlled trial that evaluated the effectiveness of routine foot trimming of heifers 3 weeks pre-calving and 100 days post-calving in reducing the first lactation lameness and improving milk production reported that preventative trimming in heifers pre- or post-calving was not associated with lameness prevalence, time to first lameness, 305-day lactation milk yield, or the type of lesions identified during dry-off claw trimming compared with only performing locomotion scoring [46]. Another study that investigated the usefulness of routine early lactation trimming in heifers reported that trimming was more effective in lame heifers at the time of trimming than in non-lame heifers in terms of milk productivity [19]. These findings suggest that dairy heifers should be selected for trimming based on the results of regular locomotion scoring, which could be more economically effective than routine trimming in this group of animals [19, 47, 48].

The average dorsal wall length in older buffaloes in the current study was 8.4 and 9.1 cm if the claws were trimmed to a step or to a point, respectively. Age-related changes in claw dimensions in cattle have been previously reported [27, 49], which is consistent with the findings of the current study. Earlier studies on Holstein Friesian cattle described the optimum dorsal wall length during the 1st step of claw trimming as 7.5 cm [50]. A recent study reported correlation between the dorsal wall length and sole thickness at the apex of the 3rd phalanx where a sole thickness of 3.8 and 4.0 mm of the medial and lateral claws, respectively were correlated with a dorsal wall length of < 7 cm. Furthermore, a sole thickness of 7 mm at the apex of 3rd phalanx was correlated with a dorsal wall length of 7.98 and 7.84 cm for the medial and lateral claws, respectively [25]. Notably, this previous study reported that the medial claw should be trimmed to approximately 1.4 mm longer than the lateral claw, which is consistent with our findings. Based on our findings, trimming to a dorsal wall length of 7.5 cm in older buffaloes will result in over-trimming in all animals. This agrees with a study by Archer et al. [27] who reported that trimming to a fixed dorsal wall length of 7.5 cm without considering the age, size of the animals and the size of the claws would result in over trimming in 97% and 96% of Holstein Friesian cattle aged ≥ 4 years and < 4 years, respectively.

## Conclusion

This study found a high prevalence of claw lesions in slaughtered buffalo. However, further studies on live animals are necessary to fully understand the impact of lameness on dairy buffaloes in Egypt. As this was an abattoir-based study, we could not elucidate the clinical significance of the identified lesions. The findings of the current study suggest a lack of routine trimming in this buffalo population, which could be due to the perceived reduced importance of routine trimming, or the less cooperative nature of this species compared with cattle. The study also provides recommendations on the minimal dorsal wall length during trimming in two age groups and reported poor to moderate agreement between ultrasonography and CT.

## Methods

### Abattoir visits and claw examination

Abattoir visits were arranged with abattoir managers, and dates for each visit were agreed upon in advance. Because buffalo feet are expensive edible carcass parts in Egypt, and the project was not funded to purchase feet for claw examination, all examinations were performed within abattoirs. Brief trimming was performed for each examined claw using a hoof knife and hoof nippers, and lesions were recorded on an examination sheet (Additional file 1). Because most lesions causing lameness in farm animals are associated with hind feet [51], only hind feet were examined in the current study. All examinations were performed by a single operator (the first author) to ensure consistency. An assistant was assigned to each visit to help record the lesions identified during the examination. Pictorial descriptions and definitions of the claw lesions were obtained from the ICAR Claw Health Atlas [52]. The slaughter law in Egypt prohibits the routine slaughtering of female buffaloes before the age of five. Therefore, two age groups were included in this part of the study: fattening buffaloes were males aged 2–3 years, and culled adult female buffaloes over 5 years of age. Sample size calculations to detect the presence of at least one claw lesion in 30% of the examined feet indicated that 300 feet were required to be examined in the study, assuming a buffalo population of 4000 would be sent for slaughter at the study area during the study period and a 95% confidence level. Sample size calculations were performed using the Epi Info software (Centre for Disease Control (CDC), Atlanta, Georgia, USA).

### Claw measurement study

Feet from two age groups were included in this study. These included apparently healthy feet purchased from government-controlled abattoirs, which belonged to adult female buffaloes over 5 years of age (*n* = 12), and apparently healthy feet collected from small private non-government-controlled abattoirs which belonged to young female buffaloes approximately 2–3 years of age (*n* = 14). These young female buffaloes were slaughtered for reasons exempt from the female age restriction (failed to conceive, clinical emergency such as upper limb fractures). Following slaughter and exsanguination, the hind feet were separated at the tarsometatarsal joints and transported to the laboratory, where they were stored at -20 °C until further ultrasonography and CT examinations.

### Ultrasonography examination

Prior to the ultrasonographic evaluation, all feet were thawed overnight at room temperature. Brief trimming was performed using a hoof knife to remove dirt and any apparently loose horn, which might have created air pockets impeding the ultrasound beam transmission. Measurements were performed using a veterinary ultrasound machine and a multifrequency linear rectal probe (Sonoscape A5 ultrasonography machine, SonoScape, China). The probe frequency was set to 6–7 MHz for most examinations, and the focus and gain were adjusted until an image of optimum quality was obtained. The probe frequency was decreased to 4 MHz in claws with greater sole thickness to obtain an image of optimum quality. The solar surface of the claws was not trimmed flat before the ultrasound scan. Therefore, a copious amount of coupling gel was applied to fill any concavity on the solar surface to ensure proper probe contact with the claw horn. The solar surface of each claw was scanned along an imaginary mid-longitudinal line from the apex of the claw to the heel region. The thicknesses of the sole and corium were measured at three locations. A location that was perpendicular to the apical margin of the third phalanx (M1), a location opposite to the deepest concavity the 3rd phalanx (M2) and a location that was opposite to the flexor tubercle of the 3rd phalanx (Fig. 2) [44]. Measurements were performed in triplicate at each of the three sites.

### Computed tomography examination

Thawed distal hind limbs underwent CT examination using an Optima CT660 clinical CT scanner (GE Medical Systems) at 120 kV and 50 mA. Scans were taken at slice thickness and spacing between slices of 0.62 mm resulting in approximately 700 images per leg (from proximal metatarsal to the tip of the claws). Images were reconstructed and visualized using RadiAnt DICOM Viewer program (Medixant, Poznan, Poland). Images were reconstructed in the axial, coronal, and sagittal planes, and approximately midsagittal images were used to obtain measurements. Lateral and medial claws were evaluated. The images were calibrated to represent the actual measurements of the specimens. Measurements were taken at the same M1, M2, and M3 sites as in the ultrasonographic examination. Additionally, dorsal wall thickness (M4) was evaluated at three different sites along the dorsal surface of the 3rd phalanx. Internal wall length was measured as the distance from the proximal limit of the wall to the tip of the corium distally (M5) [27]. Figure 3. shows an approximately midsagittal CT image with the sites of the measurements identified. The claw angle was measured as the angle formed by two straight lines, one on the dorsal surface of the wall and the other on the ground surface of the sole (Fig. 4).

The minimum recommended external dorsal wall length for each claw was estimated by adjusting the internal dorsal wall length, considering the value of the claw angle, dorsal wall thickness, and recommended minimum sole thickness of 5 mm at the tip of the 3rd phalanx [27, 49]. Trigonometry was used to perform calculations for each of the claws included in the study (Fig. 4).

### Statistical methods

The results of the claw examination were transferred to a custom-built Microsoft Access database. The data were exported to a Microsoft Excel spreadsheet for statistical analysis. The number, proportion, and 95% confidence interval (CI) of the proportions were calculated for each type of claw lesions included in the examination sheet (asymmetric claws, concave dorsal wall, corkscrew claws, scissor claws, digital dermatitis, interdigital/superficial dermatitis, axial horn fissure, heel horn erosion, horizontal fissure, vertical fissure, interdigital hyperplasia, interdigital phlegmon, diffuse sole haemorrhage, circumscribed sole haemorrhage, coronary swelling, sole ulcer, heel ulcer, toe ulcer, toe necrosis, thin sole, white line fissure, white line abscess, double sole). The relationship between buffalo sex and claw lesions identified at a proportion of ≥ 5% was examined using multiple correspondence analysis (MCA). The first two dimensions resulting from the MCA were plotted. The sex of buffaloes was used as a proxy for age, where female buffaloes were over five years of age and male buffaloes were approximately 2–3 years of age. The statistical package FactoMineR [53] was used to conduct MCA. Summary statistics (mean, standard deviation, median, interquartile range) were calculated for each claw measurement obtained from ultrasonography and CT examinations of the feet. Measurements obtained from the claws of the older buffaloes (> 5 years of age) and buffalo heifers are presented separately. The Shapiro–Wilk test was used to assess whether a measurement followed a normal distribution. The agreement between measurements (corium and sole thickness at M1, M2, and M3 sites) obtained using ultrasonography and CT examinations was assessed using the Passing–Bablok regression [54] and calculation of the intraclass correlation coefficient for agreement. The statistical packages mcr [55] and irr [56] were used to conduct the Passing–Bablok regression and to calculate the ICC for agreement, respectively. All the analyses were performed in R software version 4.2.2 [57].

### Supplementary Information

**Additional file 1. **Claw examination sheet.

## Data Availability

The datasets generated during and/or analysed during the current study are available in the figshare repository;  10.6084/m9.figshare.23566278.
