# Supplementary material for: An abattoir study of the prevalence of foot lesions and claw measurements in water buffalo in Egypt
Source: BMC Vet Res. 2024 Jan 20;20:29. doi: 10.1186/s12917-024-03877-4 (PMC10799483; doi:10.1186/s12917-024-03877-4)
Supplement: Supplementary file 1 — Additional file 1. Claw examination sheet. [file 12917_2024_3877_MOESM1_ESM.pdf]

Additional file 1: Claw examination sheet.

Abattoir: ___________________________ **Exam date:** _____________________ **Time:** _______________

Examiner: __________________________ **Assistant:** ______________________ **Species:** ☐ Buffalo ☐ Cattle

Sex: ☐ Female ☐ Male

Foot 1

Mark the drawings below with identified lesions.

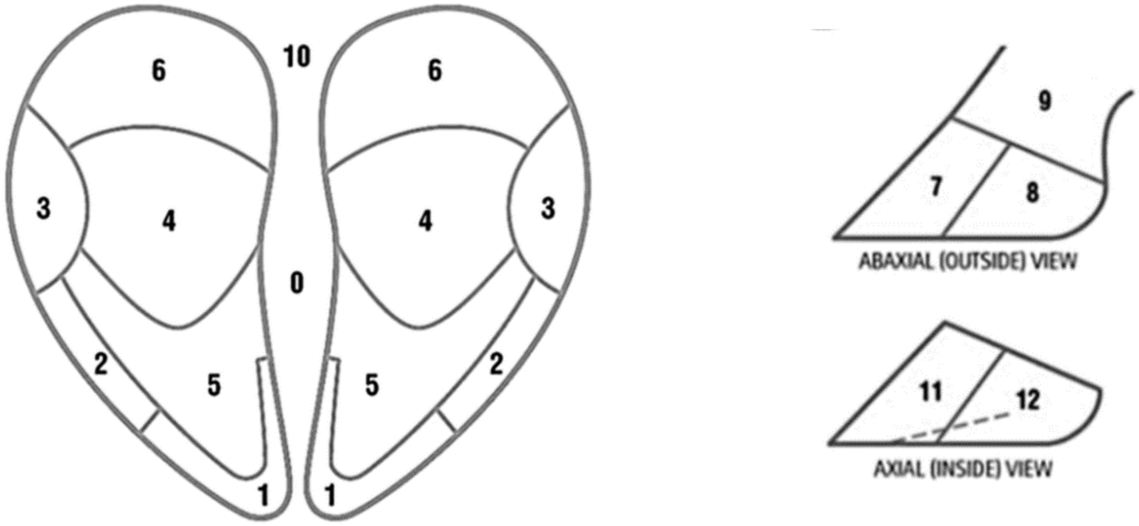

Claw diagram showing claw zones used in recording lesions (Figure courtesy of Zinpro® Corporation, Eden Prairie, MN, USA)

List of lesions identified.

Lesion	Absent/present	Lesion	Present/Absent
Asymmetric claws------------------	☐	Swelling of coronet and/or bulb	☐
Concave dorsal wall----------------	☐M ☐L	Sole ulcer ---------------------------	☐M ☐L
Corkscrew claws-------------------	☐M ☐L	Toe ulcer ----------------------------	☐M ☐L
scissor claws------------------------	☐	Heel ulcer---------------------------	☐M ☐L
Digital dermatitis------------------	☐	Toe necrosis------------------------	☐M ☐L
Interdigital/superficial dermatitis	☐	Thin sole ----------------------------	☐M ☐L
Axial horn fissure------------------	☐M ☐L	White line fissure ------------------	☐M ☐L
Heel horn erosion------------------	☐	White line abscess-----------------	☐M ☐L
Horizontal fissure ------------------	☐M ☐L	Double sole-------------------------	☐M ☐L
Vertical fissure ---------------------	☐M ☐L		
Interdigital hyperplasia -----------	☐		
Interdigital phlegmon -------------	☐		
Sole haemorrhage diffused form	☐M ☐L		
Sole haemorrhage circumscribed from	☐M ☐L		

Foot 2

Mark the drawings below with identified lesions.

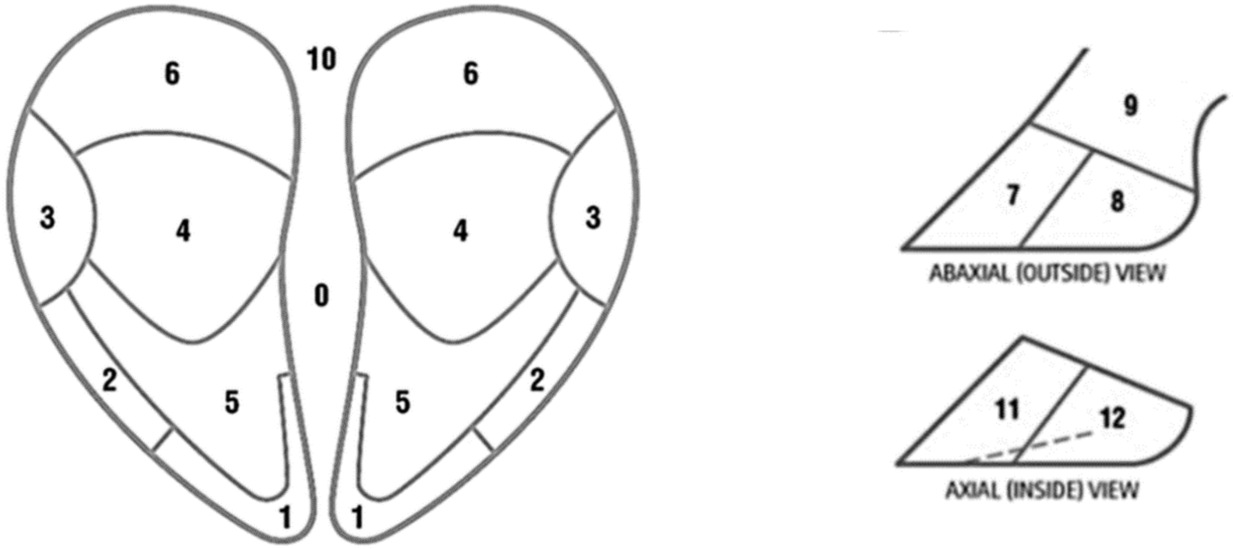

Claw diagram showing claw zones used in recording lesions (Figure courtesy of Zinpro® Corporation, Eden Prairie, MN, USA)

List of lesions identified.

Lesion	Absent/present	Lesion	Present/Absent
Asymmetric claws------------------	☐	Swelling of coronet and/or bulb	☐
Concave dorsal wall----------------	☐M ☐L	Sole ulcer ---------------------------	☐M ☐L
Corkscrew claws--------------------	☐M ☐L	Toe ulcer ----------------------------	☐M ☐L
scissor claws------------------------	☐	Heel ulcer---------------------------	☐M ☐L
Digital dermatitis-------------------	☐	Toe necrosis------------------------	☐M ☐L
Interdigital/superficial dermatitis	☐	Thin sole ----------------------------	☐M ☐L
Axial horn fissure-------------------	☐M ☐L	White line fissure -----------------	☐M ☐L
Heel horn erosion ------------------	☐	White line abscess-----------------	☐M ☐L
Horizontal fissure ------------------	☐M ☐L	Double sole-------------------------	☐M ☐L
Vertical fissure----------------------	☐M ☐L		
Interdigital hyperplasia -----------	☐		
Interdigital phlegmon -------------	☐		
Sole haemorrhage diffused form	☐M ☐L		
Sole haemorrhage circumscribed from	☐M ☐L		
